# A bio-inspired bistable recurrent cell allows for long-lasting memory

**DOI:** 10.1371/journal.pone.0252676

**Published:** 2021-06-08

**Authors:** Nicolas Vecoven, Damien Ernst, Guillaume Drion

**Affiliations:** Montefiore Institue, University of Liège, Liège, Belgium; Wroclaw University of Science and Technology, POLAND

## Abstract

Recurrent neural networks (RNNs) provide state-of-the-art performances in a wide variety of tasks that require memory. These performances can often be achieved thanks to gated recurrent cells such as gated recurrent units (GRU) and long short-term memory (LSTM). Standard gated cells share a layer internal state to store information at the network level, and long term memory is shaped by network-wide recurrent connection weights. Biological neurons on the other hand are capable of holding information at the cellular level for an arbitrary long amount of time through a process called bistability. Through bistability, cells can stabilize to different stable states depending on their own past state and inputs, which permits the durable storing of past information in neuron state. In this work, we take inspiration from biological neuron bistability to embed RNNs with long-lasting memory at the cellular level. This leads to the introduction of a new bistable biologically-inspired recurrent cell that is shown to strongly improves RNN performance on time-series which require very long memory, despite using only cellular connections (all recurrent connections are from neurons to themselves, i.e. a neuron state is not influenced by the state of other neurons). Furthermore, equipping this cell with recurrent neuromodulation permits to link them to standard GRU cells, taking a step towards the biological plausibility of GRU. With this link, this work paves the way for studying more complex and biologically plausible neuromodulation schemes as gating mechanisms in RNNs.

## 1 Introduction

Recurrent neural networks (RNNs) have been widely used in the past years, providing excellent performances on many problems requiring memory such as sequence to sequence modeling, speech recognition, and neural translation. These achievements are often the result of the development of the long short-term memory (LSTM [1]) and gated recurrent units (GRU [2]) recurrent cells, which allow RNNs to capture time-dependencies over long horizons. Despite all the work analyzing the performances of such cells [3], recurrent cells remain predominantly black-box models. There has been some advance in understanding the dynamical properties of RNNs as a whole from a non-linear control perspective [4], showing the importance of fixed points in trained networks. However, little has been done in understanding the underlying system of recurrent cells themselves. Rather, they have been built for their robust mathematical properties when computing gradients with back-propagation through time (BPTT), as training RNNs has been known to be difficult [5]. Research on new recurrent cells is still ongoing and, building up on LSTM and GRU, recent works have proposed other types of gated units [6–8]. In addition, an empirical search over hundreds of different gated architectures has been carried in [9]. These works showed the possibility to achieve better performances than widely-used cells such as GRUs and LSTMs by only slightly changing gating mechanisms, hinting that such small changes can have major impact on the RNN dynamics.

In parallel, there has been an increased interest in assessing the biological plausibility of neural networks. There has not only been a lot of interest in spiking neural networks [10–12], but also in reconciling more traditional deep learning models with biological plausibility [13–15]. RNNs are a promising avenue for the latter [16] as they are known to provide great performances from a deep learning point of view while theoretically allowing a discrete dynamical simulation of biological neurons.

RNNs combine simple cellular dynamics and a rich, highly recurrent network architecture. The recurrent network architecture enables the encoding of complex memory patterns in the connection weights. These memory patterns rely on global feedback interconnections of large neuronal populations. Such global feedback interconnections are difficult to tune, and can be a source of vanishing or exploding gradient during training, which is a major drawback of RNNs. In biological networks, a significant part of advanced computing is handled at the cellular level, mitigating the burden at the network level. Each neuron type can switch between several complex firing patterns, which include e.g. spiking, bursting, and bistability. In particular, bistability is the ability for a neuron to switch between two stable outputs depending on input history. It is a form of cellular memory [17].

In this work, we propose a new biologically motivated bistable recurrent cell (BRC), which embeds classical RNNs with local cellular memory rather than global network memory. More precisely, BRCs are built such that their hidden recurrent state does not directly influence other neurons (i.e. they are not recurrently connected to other cells). To make cellular bistability compatible with the RNNs feedback architecture, a BRC is constructed by taking a feedback control perspective on biological neuron excitability [18]. This approach enables the design of biologically-inspired cellular dynamics by exploiting the RNNs structure rather than through the addition of complex mathematical functions.

We show that, despite having only cellular temporal connections, BRCs provide decent performances on standard benchmarks and outperform classic RNN cells as GRUs and LSTMs on benchmarks with datasets requiring long-term memory, highlighting the importance of bistability. To further improve BRCs performances, we endow them with recurrent neuromodulation, leading to a new neuromodulated bistable recurrent cell (nBRC). We carry a thorough analysis of the performances of nBRCs against state-of-the-art cells and show that they are the top performers when long-term memory requirements are important.

## 2 Recurrent neural networks and gated recurrent units

RNNs have been widely used to tackle many problems having a temporal structure. In such problems, the relevant information can only be captured by processing observations obtained during multiple time-steps. More formally, a time-series can be defined as **X** = [**x**_0_, …, **x**_*T*_] with $T\in N_{0}$ and $x_{i}\in R^{n}$. To capture time-dependencies, RNNs maintain a recurrent hidden state whose update depends on the previous hidden state and current observation of a time-series, making them dynamical systems and allowing them to handle arbitrarily long sequences of inputs. Mathematically, RNNs maintain a hidden state **h**_*t*_ = *f*(**h**_*t*−1_, **x**_*t*_;*θ*), where **h**_0_ is a constant and *θ* are the parameters of the network. In its most standard form, an RNN updates its state as follows:

$$\begin{array}{c} h_{t}=g\left(Ux_{t}+Wh_{t-1}\right) \end{array}$$ (1)

where *g* is a standard activation function such as a sigmoid or a hyperbolic tangent. However, RNNs using Eq 1 as the update rule are known to be difficult to train on long sequences due to vanishing (or, more rarely, exploding) gradient problems. To alleviate this problem, more complex recurrent update rules have been proposed, such as LSTMs [1] and GRUs [2]. These updates allow recurrent networks to be trained on much longer sequences by using gating principles. By way of illustration, the updates related to a gated recurrent unit are

$$\begin{array}{c} \left\{ \begin{array}{c} z_{t}=\sigma \left(U_{z}x_{t}+W_{z}h_{t-1}\right)\\ r_{t}=\sigma \left(U_{r}x_{t}+W_{r}h_{t-1}\right)\\ h_{t}=z_{t}⊙h_{t-1}+\left(1-z_{t}\right)⊙\tanh\left(U_{h}x_{t}+r_{t}⊙W_{h}h_{t-1}\right) \end{array}\right. \end{array}$$ (2)

where **z** is the update gate (used to tune the update speed of the hidden state with respect to new inputs) and **r** is the reset gate (used to reset parts of the memory).

## 3 Neuronal bistability: A feedback viewpoint

Biological neurons are intrinsically dynamical systems that can exhibit a wide variety of firing patterns. In this work, we focus on the control of bistability, which corresponds to the coexistence of two stable states at the neuronal level. Bistable neurons can switch between their two stable states in response to transient inputs [17, 19], endowing them with a kind of never-fading cellular memory [17].

Complex neuron firing patterns are often modeled by systems of ordinary differential equations (ODEs). Translating ODEs into an artificial neural network algorithm often leads to mixed results due to increased complexity and the difference in modeling language. Another approach to model neuronal dynamics is to use a control systems viewpoint [18]. In this viewpoint, a neuron is modeled as a set of simple building blocks connected using a multi-scale feedback, or recurrent, interconnection pattern.

**Fig 1:**
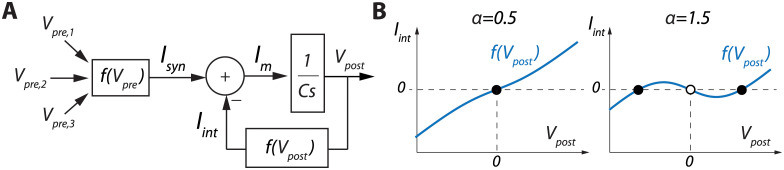
**A**. One timescale control diagram of a neuron. **B**. Plot of the function *I*_*int*_ = *V*_*post*_−*α*tanh(*V*_*post*_) for two different values of *α*. Full dots correspond to stable states, empty dots to unstable states.

A neuronal feedback diagram focusing on one time-scale, which is sufficient for bistability, is illustrated in Fig 1A. The block 1/(*Cs*) accounts for membrane integration, *C* being the membrane capacitance and *s* the complex frequency. The outputs from presynaptic neurons *V*_*pre*_ are combined at the input level to create a synaptic current *I*_*syn*_. Neuron-intrinsic dynamics are modeled by the negative feedback interconnection of a nonlinear function *I*_*int*_ = *f*(*V*_*post*_), called the IV curve in neurophysiology, which outputs an intrinsic current *I*_*int*_ that adds to *I*_*syn*_ to create the membrane current *I*_*m*_. The slope of *f*(*V*_*post*_) determines the feedback gain, a positive slope leading to negative feedback and a negative slope to positive feedback. *I*_*m*_ is then integrated by the postsynaptic neuron membrane to modify its output voltage *V*_*post*_.

The switch between monostability and bistability is achieved by shaping the nonlinear function *I*_*int*_ = *f*(*V*_*post*_) (Fig 1B). The neuron is monostable when *f*(*V*_*post*_) is monotonic of positive slope (Fig 1B, left). Its only stable state corresponds to the voltage at which *I*_*int*_ = 0 in the absence of synaptic inputs (full dot). The neuron switch to bistability through the creation of a local region of negative slope in *f*(*V*_*post*_) (Fig 1B, left). Its two stable states correspond to the voltages at which *I*_*int*_ = 0 with positive slope (full dots), separated by an unstable state where *I*_*int*_ = 0 with negative slope (empty dot). The local region of negative slope corresponds to a local positive feedback where the membrane voltage is unstable.

In biological neurons, a local positive feedback is provided by regenerative gating, such as sodium and calcium channel activation or potassium channel inactivation [19, 20]. The switch from monostability to bistability can therefore be controlled by tuning ion channel density. This property can be emulated in electrical circuits by combining transconductance amplifiers to create the function

$$\begin{array}{c} I_{int}=V_{post}-\alpha \tanh\left(V_{post}\right), \end{array}$$ (3)

where the switch from monostability to bistability is controlled by a single parameter *α* [21]. *α* models the effect of sodium or calcium channel activation, which tunes the local slope of the function, hence the local gain of the feedback loop (Fig 1B). For *α* ∈ ]0, 1] (where ]0, 1] denotes a continuous interval), which models a low sodium or calcium channel density, the function is monotonic, leading to monostability (Fig 1B, left). For *α* ∈ ]1, + ∞[, which models a high sodium or calcium channel density, a region of negative slope is created around *V*_*post*_ = 0, and the neuron becomes bistable (Fig 1B, right). This bistability leads to never-fading memory, as in the absence of significant input perturbation the system will remain indefinitely in one of the two stable states depending on the input history.

Neuronal bistability can therefore be modeled by a simple feedback system whose dynamics is tuned by a single feedback parameter *α*. This parameter can switch between monostability and bistability by tuning the shape of the feedback function *f*(*V*_*post*_), whereas neuron convergence dynamics is controlled by a single feedforward parameter *C*. In biological neurons, both these parameters can be modified dynamically by other neurons via a mechanism called neuromodulation, providing a dynamic, controllable memory at the cellular level. The key challenge is to find an appropriate mathematical representation of this mechanism to be efficiently used in artificial neural networks, and, more particularly, in RNNs.

## 4 Cellular memory, bistability and neuromodulation in RNNs

### 4.1 The bistable recurrent cell (BRC)

To model controllable bistability in RNNs, we start by drawing two main comparisons between the feedback structure Fig 1A and the GRU equations (Eq 2). First, we note that the reset gate *r* has a role that is similar to the one played by the feedback gain *α* in Eq 3. In GRU equations, *r* is the output of a sigmoid function, which implies *r* ∈ ]0, 1[. These possible values for *r* correspond to negative feedback only, which does not allow for bistability. The update gate *z*, on the other hand, has a role similar to that of the membrane capacitance *C*. Second, one can see through the matrix multiplications *W*_*z*_ **h**_*t*−1_, *W*_*r*_ **h**_*t*−1_ and *W*_*h*_ **h**_*t*−1_ that each cell uses the internal state of other neurons to compute its own state without going through synaptic connections. In biological neurons, the intrinsic dynamics defined by *I*_*int*_ is constrained to only depend on its own state *V*_*post*_, and the influence of other neurons comes only through the synaptic compartment (*I*_*syn*_), or through neuromodulation.

To enforce this cellular feedback constraint in GRU equations and to endow them with bistability, we propose to update *h*_*t*_ as follows:

$$\begin{array}{c} h_{t}=c_{t}⊙h_{t-1}+\left(1-c_{t}\right)⊙\tanh\left(Ux_{t}+a_{t}⊙h_{t-1}\right) \end{array}$$ (4)

where **a**_*t*_ = 1 + tanh(*U*_*a*_ **x**_*t*_ + **w**_*a*_⊙**h**_*t*−1_) and **c**_*t*_ = *σ*(*U*_*c*_ **x**_*t*_ + **w**_*c*_⊙**h**_*t*−1_). **a**_*t*_ corresponds to the feedback parameter *α*, with **a**_*t*_ ∈ ]0, 2[ (as tanh(⋅)∈] − 1, 1[). **c**_*t*_ corresponds to the update gate in GRU and plays the role of the membrane capacitance *C*, determining the convergence dynamics of the neuron. We call this updated cell the bistable recurrent cell (BRC).

**Fig 2:**
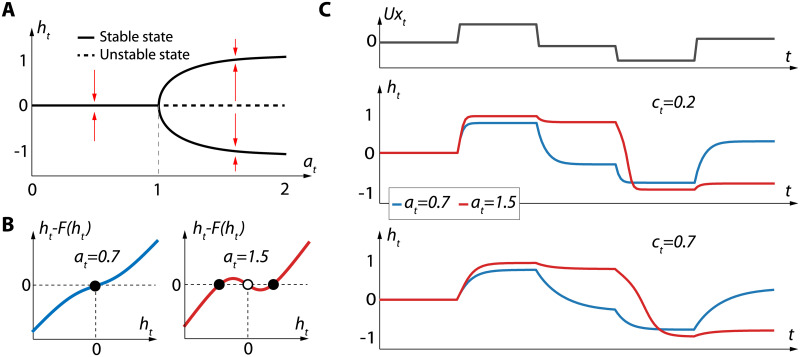
**A**. Bifurcation diagram of Eq 4 for *U* **x**_*t*_ = 0. **B**. Plots of the function *h*_*t*_ − *F*(*h*_*t*_) for two values of *a*_*t*_, where *F*(*h*_*t*_) = *c*_*t*_ *h*_*t*_ + (1 − *c*_*t*_)tanh(*a*_*t*_ *h*_*t*_) is the right hand side of Eq 4 with *x*_*t*_ = 0. Full dots correspond to stable states, empty dots to unstable states. **C**. Response of BRC to an input time-series for different values of *a*_*t*_ and *c*_*t*_.

The main differences between a BRC and a GRU are twofold. First, each neuron has its own internal state **h**_*t*_ that is not directly affected by the internal state of the other neurons. Indeed, due to the four instances of **h**_*t*−1_ coming from Hadamard products, the only temporal connections existing in layers of BRC are from neurons to themselves. This enforces the memory to be only cellular. Second, the feedback parameter **a**_*t*_ is allowed to take a value in the range ]0, 2[ rather than ]0, 1[. This allows the cell to switch between monostability (*a* ≤ 1) and bistability (*a* > 1) (Fig 2A and 2B). The proof of this switch is provided in Appendix A.

It is important to note that the parameters **a**_*t*_ and **c**_*t*_ are dynamic. **a**_*t*_ and **c**_*t*_ are neuromodulated by the previous layer, that is, their value depends on the output of other neurons. Tests were carried with **a** and **c** as parameters learned by stochastic gradient descent, which resulted in lack of representational power, leading to the need for neuromodulation. This neuromodulation scheme was the most evident as it maintains the cellular memory constraint and leads to the most similar update rule with respect to standard recurrent cells (Eq 2). However, as will be discussed later, other neuromodulation schemes can be thought of.

Likewise, from a neuroscience perspective, **a**_*t*_ could well be greater than 2. Limiting the range of **a**_*t*_ to ]0, 2[ was made for numerical stability and for symmetry between the range of bistable and monostable neurons. We argue that this is not an issue as, for a value of **a**_*t*_ greater than 1.5, the dynamics of the neurons become very similar (as suggested in Fig 2A).

Fig 2C shows the dynamics of a BRC with respect to *a*_*t*_ and *c*_*t*_. For *a*_*t*_ < 1, the cell exhibits a classical monostable behavior, relaxing to the 0 stable state in the absence of inputs (blue curves in Fig 2C). On the other hand, a bistable behavior can be observed for *a*_*t*_ > 1: the cells can either stabilize on an upper stable state or a lower stable state depending on past inputs (red curves in Fig 2C). Since these upper and lower stable states do not correspond to an *h*_*t*_ which is equal to 0, they can be associated with cellular memory that never fades over time. Furthermore, Fig 2 also illustrates that neuron convergence dynamics depend on the value of *c*.

### 4.2 The recurrently neuromodulated bistable recurrent cell (nBRC)

To further improve the performance of BRC, one can relax the cellular memory constraint. By creating a dependency of *a*_*t*_ and *c*_*t*_ on the output of other neurons of the layer, one can build a kind of recurrent layer-wise neuromodulation. We refer to this modified version of a BRC as an nBRC, standing for recurrently neuromodulated BRC. The update rule for the nBRC is the same as for BRC, and follows Eq 4. The difference comes in the computation of *a*_*t*_ and *c*_*t*_, which are neuromodulated as follows:

$$\begin{array}{c} \left\{ \begin{array}{c} a_{t}=1+\tanh\left(U_{a}x_{t}+W_{a}h_{t-1}\right)\\ c_{t}=\sigma \left(U_{c}x_{t}+W_{c}h_{t-1}\right) \end{array}\right. \end{array}$$ (5)

The update rule of nBRCs being that of BRCs (Eq 4), bistable properties are maintained and hence the possibility of a cellular memory that does not fade over time. However, the new recurrent neuromodulation scheme adds a type of network memory on top of the cellular memory.

This recurrent neuromodulation scheme brings the update rule even closer to standard GRU. This is highlighted when comparing Eqs 2 and 4 with parameters neuromodulated following Eq 5. We stress that, as opposed to GRUs, bistability is still ensured through *a*_*t*_ belonging to ]0, 2[ (thus the possibility to be greater than 1). A relaxed cellular memory constraint is also ensured, as each neuron past state *h*_*t*−1_ only directly influences its own current state and not the state of other neurons of the layer (Hadamard product on the **h**_*t*_ update in Eq 4). This is important for numerical stability as the introduction of a cellular positive feedback for bistability leads to global instability if the update is computed using other neurons states directly (as it is done in the classical GRU update, see the matrix multiplication *W*_*h*_ **h**_*t*−1_ in Eq 2).

Finally, let us note that to be consistent with the biological model presented in Section 3, Eq 5 should be interpreted as a way to represent a neuromodulation mechanism of a neuron by those from its own layer and the layer that precedes. Hence, the possible analogy between gates *z* and *r* in GRUs and neuromodulation. In this respect, studying the introduction of new types of gates based on more biological plausible neuromodulation architectures would certainly be interesting.

## 5 Analysis of BRC and nBRC performance

To demonstrate the impact of bistability in RNNs we tackle four problems. The first is a one-dimensional toy problem, the second is a two-dimensional denoising problem, the third is the permuted sequential MNIST problem and the fourth is a variation of the third benchmark. All benchmarks are related to a supervised setting. The network is presented with a time-series and is asked to output a prediction (regression for the first two benchmarks and classification for the others) after having received the last element(s) of the time-series **x**_*T*_. Note that for the second benchmark the regression is carried over multiple time-steps (sequence-to-sequence) whereas, this prediction is given in a single time-step after receiving **x**_*T*_ for the other benchmarks. We first show that the introduction of bistability in recurrent cells is especially useful for datasets in which only time-series with long time-dependencies are available. We achieve this by comparing results of BRC and nBRC to other recurrent cells. We use LSTMs [1] and GRUs [2] as a baseline since they have already been established. We also compare to two other cells (GORUs [8] and LMUs [22]) which were developed to improve performance over GRUs and LSTMs and use them as our state-of-the-art comparison. Finally, we also take a look at the dynamics inside the nBRC neurons in the context of the denoising benchmark and show that bistability is heavily used by the neural network.

### 5.1 Results

For the first two problems, training sets comprise 40000 samples and performances are evaluated on test sets generated with 50000 samples. For the permuted MNIST benchmarks, the standard train and test sets are used. All averages and standard deviations reported were computed over three different seeds. We found that there were only minor variations in between runs, and thus believe that three runs are sufficient to capture the performance of the different architectures. For all benchmarks, networks are composed of two layers of 128 neurons. Different recurrent cells are always tested on similar networks (i.e. same number of layers/neurons). We used the tensorflow [23] implementation of GRUs. Finally, the ADAM optimizer with a learning rate of 1*e*^−3^ is used for training all networks, with a mini-batch size of 100. The source code for carrying out the experiments is available at https://github.com/nvecoven/BRC. All networks are trained for 50 epochs (which has proven to be enough to reach convergence on these particular benchmarks).

#### 5.1.1 Copy first input benchmark

**Fig 3:**
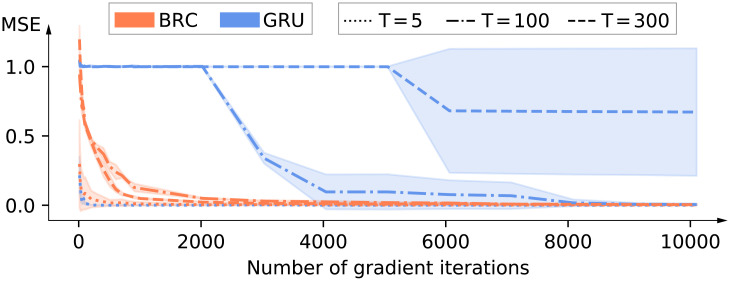
Evolution of the average mean-square error (± standard deviation) over three runs on the copy input benchmark for GRU and BRC and for different values of *T*.

In this benchmark, the network is presented with a one-dimensional time-series of *T* time-steps where $x_{t}∼N\left(0,1\right),\;\forall t\in T$. After receiving *x*_*T*_, the network output value should approximate *x*_0_, a task that is well suited for capturing their capacity to learn long temporal dependencies if *T* is large. Note that this benchmark also requires the ability to filter irrelevant signals as, after time-step 0, the networks are continuously presented with noisy inputs that they must learn to ignore. The mean square error on the test set is shown for different values of *T* in Table 1. In this benchmark, one can see the limitation of LSTMs and GRUs when *T* becomes large (this is shown in particular for GRUs on Fig 3), as they are unable to beat random guessing performances (which would be equal to 1 in this setting. Indeed, as *x*_0_ is sampled from a normal distribution N(0,1), guessing 0 would lead to the lowest error which would on average be equal to the standard deviation). Furthermore, we see that the gated orthogonal version of GRUs (GORUs) achieves better performances than GRU cells, as expected. We also see that thanks to bistability, nBRCs and BRCs are able to learn effectively and achieve similar performances to those of LMUs.

**Table 1:** Mean square error (± standard deviation) of different architectures on the test set for the copy input benchmark. Results are shown after 50 epochs and for different values of *T*.

*T*	BRC	NBRC	GORU	LSTMCell	GRUCell	LMU
5	0.005±0.001	0.000±0.000	0.000±0.000	0.000±0.000	0.000±0.000	0.000±0.000
50	0.082±0.027	0.002±0.000	0.019±0.009	0.000±0.000	0.997±0.005	0.000±0.000
300	0.086±0.014	0.010±0.003	0.308±0.050	1.002±0.009	0.876±0.190	0.000±0.000
600	0.099±0.029	0.009±0.002	0.323±0.068	0.989±0.008	0.999±0.017	0.002±0.001

#### 5.1.2 Denoising benchmark

The copy input benchmark is interesting as a means to highlight the memorisation capacity of the recurrent neural network, but it does not tackle its ability to successfully exploit complex relationships between different elements of the input signal to predict the output. In the denoising benchmark, the network is presented with a two-dimensional time-series of *T* time-steps. Five different time-steps *t*_1_, …, *t*_5_, for which data should be remembered, are sampled uniformly in {0, …, *T* − *N*} with *N* ∈ {5, …, *T* − 4} and are communicated to the network through the first dimension of the time-series by setting **x**_*t*_[1] = 1 if *t* ∈ {*t*_1_, …, *t*_5_}, **x**_*t*_[1] = 0 if *t* = *T* − 4 and **x**_*t*_[1] = −1 otherwise. Note that the parameter *N* controls the length of the forgetting period as it forces the relevant inputs to be in the first *T* − *N* time-steps. This ensures that *t*_*x*_ < *T* − *N*, Â ∀*x* ∈ {1, …, 5}. Also note that this dimension can be used by the network to know its prediction is now starting to influence the loss (whenever **x**_*t*_[1] = 0).

The second dimension is a data-stream, generated as for the copy first input benchmark, that is $x_{t}\left[2\right]∼N\left(0,1\right),\forall t\in \left\{0,\ldots ,T-4\right\}$ and **x**_*t*_[2] = 0, ∀*t* ∈ {*T* − 4, …, *T*}. At time-step *T* − 4, the network is asked to output $x_{t_{1}}\left[2\right]$, at time-step *T* − 3 the network is asked to output $x_{t_{2}}\left[2\right]$ and so on until time-step *T* at which it should output $x_{t_{5}}\left[2\right]$. The mean squared error is averaged over the five values. That is, the error on the prediction is equal to $\sum_{i=1}^{5}\frac{{\left(x_{t_{i}}\left[2\right]-O_{T-5+i}\right)}^{2}}{5}$ with **O**_**x**_ the output of the neural network at time-step *x*.

As one can see in Table 2 (generated with *T* = 400 and two different values of *N*), for *N* = 200 only bistable cells are able to achieve good performances.

**Table 2:** Mean square error (± standard deviation) of different architectures on the denoising benchmark’s test set. Results are shown with and without constraint on the location of relevant inputs and after 50 epochs. Relevant inputs cannot appear in the *N* last time-steps, that is **x**_*t*_[1] = −1, ∀*t* > (*T* − *N*). In this experiment, results were obtained with *T* = 400.

*N*	BRC	NBRC	GORU	LSTM	GRU	LMU
5	0.579±0.033	0.016±0.003	0.000±0.000	0.655±0.463	0.001±0.000	1.004±0.006
200	0.614±0.119	0.071±0.078	1.004±0.003	0.996±0.005	0.995±0.003	1.000±0.003

#### 5.1.3 Permuted sequential MNIST

In this benchmark, the network is presented with the MNIST images, where pixels are shown, one by one, as a time-series. It differs from the regular sequential MNIST in that pixels are shuffled, with the result that they are not shown in top-left to bottom-right order. This benchmark is known to be a more complex challenge than the regular one. Indeed, shuffling makes time-dependencies more complex by introducing lag in between pixels that are close together in the image, thus making structure in the time-serie harder to find. MNIST images are comprised of 784 pixels (28 by 28), requiring dynamics over hundreds of time-steps to be learned. Table 3 shows that bistability does not hinder performances when compared to GRU cells, even for more complex standard benchmarks, in which specific long-term memory is not required. In this case, only LMUs provide significantly better performance than nBRCs, which are otherwise competitive with all other cell types. This table also shows that the introduction of cellular connections does not hinder much the representational power of the cell.

**Table 3:** Overall accuracy and macro-averaged F1-score (± standard deviation) on the permuted sequential MNIST benchmark’s test set after 50 epochs and for different cell types.

	BRC	NBRC	GORU	LSTMCell	GRUCell	LMU
Acc.	0.662±0.007	0.908±0.006	0.902±0.004	0.910±0.002	0.908±0.004	0.969±0.001
F1	0.655±0.007	0.906±0.005	0.897±0.008	0.907±0.003	0.902±0.006	0.965±0.002

#### 5.1.4 Permuted line-sequential MNIST

In this benchmark, we use the same permutation of pixels in the MNIST images as for the previous benchmark. We then feed the pixels to the RNNs line by line, thus allowing one to test the networks with a higher input dimension (28 in this case). Furthermore, to highlight once again the interest of bistability, we add *N* black lines at the end of the image. This has the effect of a forgetting period, as any relevant information for predicting the output will be farther from the prediction time-step in the time-serie. As for the copy input benchmark, we see on Table 4 that only bistable cells and LMUs are able to tackle this problem correctly.

**Table 4:** Overall accuracy and macro-averaged F1-score (± standard deviation) on permuted sequential-line MNIST test set after 50 epochs for different architectures. Images are fed to the recurrent network line by line and *N* black lines are added at the bottom of the image after permutation. We note that when *N* equals 72(472) the resulting image has 100(500) lines.

	*N*	BRC	NBRC	GORU	LSTMCell	GRUCell	LMU
Acc.	72	0.968±0.001	0.973±0.001	0.977±0.000	0.977±0.002	0.977±0.002	0.969±0.001
F1	72	0.967±0.001	0.972±0.001	0.972±0.000	0.976±0.002	0.974±0.001	0.941±0.002
Acc.	472	0.960±0.001	0.972±0.002	0.198±0.021	0.562±0.328	0.591±0.388	0.961±0.003
F1	472	0.956±0.001	0.972±0.002	0.083±0.0031	0.454±0.453	0.495±0.477	0.898±0.013

#### 5.1.5 Permuted variable-line-sequential MNIST

Finally, to test the capacity of the network on variable-length sequences, we also test a variation of this benchmark, which we call permuted variable-sequential-line MNIST. In this variation, a random number *N* of black lines are added to the image (after permutation of the pixels) for each sample, where *N* is sampled uniformly in U{0,…,X}. Additionally, all pixels of the last line are assigned a high positive value (greater than the value corresponding to that of a white pixel, so that this line can never appear in a standard image). This line can be used by the network for it to know it should output the class of the image for that particular time-step. We note that in this benchmark, samples are of variable lengths. In this case (Table 5, results are more similar to those obtained in the denoising benchmark.

**Table 5:** Overall accuracy and macro-averaged F1-score (± standard deviation) on the permuted variable-sequential-line MNIST test set after 50 epochs for different architectures. Images are fed to the recurrent network line by line and N∼U{0,…,X} black lines are added at the bottom of the image after permutation.

	*X*	BRC	NBRC	GORU	LSTMCell	GRUCell	LMU
Acc.	472	0.958±0.002	0.970±0.001	0.148±0.015	0.630±0.318	0.540±0.426	0.180±0.002
F1	472	0.954±0.000	0.967±0.001	0.022±0.001	0.491±0.465	0.451±0.474	0.062±0.036

### 5.2 Analysis of nBRC dynamic behavior

**Fig 4:**
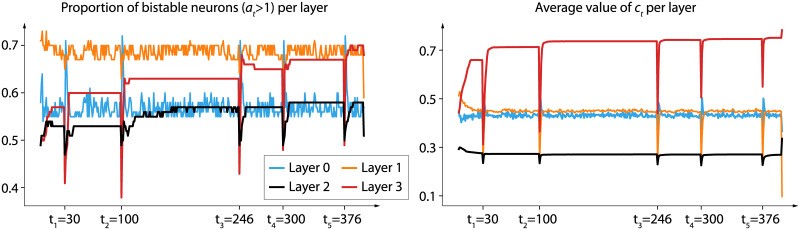
Representation of the nBRC parameters, per layer, of a recurrent neural network (with 4 layers of 100 neurons each), when shown a time-series of the denoising benchmark (*T* = 400, *N* = 0). Layer numbering increases as layers get deeper (i.e. layer i corresponds to the ith layer of the network). The 5 time-steps at which a relevant input is shown to the model are clearly distinguishable by the behaviour of those measures alone.

Until now, we have looked at the learning performances of bistable recurrent cells. It is, however, interesting to take a deeper look at the dynamics of such cells to understand whether or not bistability is used by the network. To this end, we pick a random time-series from the denoising benchmark and analyse some properties of *a*_*t*_ and *c*_*t*_. For this analysis, we train a network with 4 layers of 100 neurons each, allowing for the analysis of a deeper network as compared to those used in the benchmarks. Note that the performances of this network are similar to those reported in Table 2. Fig 4 shows the proportion of bistable cells per layer and the average value of *e*_*t*_ per layer. The dynamics of the parameters show that they are well used by the network, and three main observations should be made. First, as relevant inputs are presented to the network, the proportion of bistable neurons tends to increase in layers 2 and 3, effectively storing information and thus confirming the interest of introducing bistability for long-term memory. As more information needs to be stored, the network leverages the power of bistability by increasing the number of bistable neurons. Second, as relevant inputs are presented to the network, the average value *c*_*t*_ tends to increase in layer 3, effectively making the network less and less sensitive to new inputs. Third, one can observe a transition regime when a relevant input is shown. Indeed, there is a high decrease in the average value of *c*_*t*_, effectively making the network extremely sensitive to the current input, which allows for its efficient memorization.

## 6 Conclusion

In this paper, we introduced two new important concepts from the biological brain into recurrent neural networks: cellular memory and bistability. This led to the development of two new cells, called the Bistable Recurrent Cell (BRC) and recurrently neuromodulated Bistable Recurrent Cell (nBRC) that proved to be very efficient on several datasets requiring long-term memory and on which the performances of classical recurrent cells such as GRUs and LSTMS were poor. Furthermore, through the similarities between nBRCs and standard GRUs, we highlight that gating mechanisms can be linked to biological neuromodulation.

As future work, it would be of interest to study more complex and biologically plausible neuromodulation schemes and identify what types of new, gated architectures could emerge from them. A well biologically-motivated example of this would be the use of a neuromodulatory network [24] to compute gate activations.

Furthermore, we note that even though we focused on supervised benchmarks in the context of this paper, bistable cells might be of great use for reinforcement learning (RL), and more precisely for RL problems with sparse environments. These problems have been known to be extremely hard to solve, on one hand due to the difficulty of exploration and on the other hand due to the difficulty of remembering relevant information across large periods of time-steps. Bistable cells are a promising avenue for solving the latter, and might be a worthwhile path to explore.

## 7 Appendix

### A Proof of bistability for BRC and nBRC for *a*_*t*_ > 1

**Theorem A.1.** *The system defined by the equation*

$$\begin{array}{c} h_{t}=ch_{t-1}+\left(1-c\right)\tanh\left(Ux_{t}+ah_{t-1}\right)=F\left(h_{t-1}\right) \end{array}$$ (6)

*with c* ∈ [0, 1] *is monostable for a* ∈ [0, 1[ *and bistable for a* > 1 *in some finite range of Ux*_*t*_ *centered around x*_*t*_ = 0.

*Proof*. We can show that the system undergoes a supercritical pitchfork bifurcation at the equilibrium point (*x*_0_, *h*_0_) = (0, 0) for *a* = *a*_*pf*_ = 1 by verifying the conditions

$$\begin{array}{cc} & G\left(h_{0}\right){|}_{a_{pf}}=\frac{dG(h_{t})}{dh_{t}}{|}_{h_{0},a_{pf}}=\frac{d^{2}G\left(h_{t}\right)}{dh_{t}^{2}}{|}_{h_{0},a_{pf}}=\frac{dG(h_{t})}{da}{|}_{h_{0},a_{pf}}=0 \end{array}$$ (7)

$$\begin{array}{cc} & \frac{d^{3}G\left(h_{t}\right)}{dh_{t}^{3}}{|}_{h_{0},a_{pf}}>0,\frac{d^{2}G\left(h_{t}\right)}{dh_{t}da}{|}_{h_{0},a_{pf}}<0 \end{array}$$ (8)

where *G*(*h*_*t*_) = *h*_*t*_ − *F*(*h*_*t*_) [25]. This gives

$$\begin{array}{ccc} G\left(h_{0}\right){|}_{a_{pf}} & = & \left(1-c\right)(h_{0}-\tanh\left(a_{pf}h_{0}\right))=0, \end{array}$$ (9)

$$\begin{array}{ccc} \frac{dG(h_{t})}{dh_{t}}{|}_{h_{0},a_{pf}} & = & \left(1-c\right)\left(a_{pf}\left(\tanh^{2}\left(a_{pf}h_{0}\right)-1\right)+1\right)=\left(1-c\right)\left(1-a_{pf}\right)=0, \end{array}$$ (10)

$$\begin{array}{ccc} \frac{d^{2}G\left(h_{t}\right)}{dh_{t}^{2}}{|}_{h_{0},a_{pf}} & = & \left(1-c\right)2a_{pf}^{2}\tanh\left(a_{pf}h_{0}\right)\left(1-\tanh^{2}\left(a_{pf}h_{0}\right)\right)=0, \end{array}$$ (11)

$$\begin{array}{ccc} \frac{dG(h_{t})}{da}{|}_{h_{0},a_{pf}} & = & \left(1-c\right)h_{0}\left(\tanh{\left(a_{pf}h_{0}\right)}^{2}-1\right)=0, \end{array}$$ (12)

$$\begin{array}{cc} \frac{d^{3}G\left(h_{t}\right)}{dh_{t}^{3}}{|}_{h_{0},a_{pf}} & =\left(1-c\right)*(2a^{3}{\left(\tanh^{2}\left(a_{pf}h_{0}\right)-1\right)}^{2}+4a_{pf}^{3}\tanh^{2}\left(a_{pf}h_{0}\right)\left(\tanh^{2}\left(a_{pf}h_{0}\right)-1\right))\\ & =2(1-c)>0, \end{array}$$ (13)

$$\begin{array}{cc} \frac{d^{2}G\left(h_{t}\right)}{dh_{t}da}{|}_{h_{0},a_{pf}} & =\left(1-c\right)(\left(\tanh^{2}\left(a_{pf}h_{0}\right)-1\right)+2a_{pf}h_{0}\tanh\left(a_{pf}h_{0}\right)\left(1-\tanh^{2}\left(a_{pf}h_{0}\right)\right))\\ & =c-1<0. \end{array}$$ (14)

The stability of (*x*_0_, *h*_0_) for *a* ≠ 1 can be assessed by studying the linearized system

$$\begin{array}{c} h_{t}=\frac{dF(h_{t})}{dh_{t}}{|}_{h_{0}}h_{t-1}. \end{array}$$ (15)

The equilibrium point is stable if *dF*(*h*_*t*_)/*dh*_*t*_ ∈ [0, 1[, singular if *dF*(*h*_*t*_)/*dh*_*t*_ = 1, and unstable if *dF*(*h*_*t*_)/*dh*_*t*_ ∈ ]1, + ∞[. We have

$$\begin{array}{ccc} \frac{dF(h_{t})}{dh_{t}}{|}_{h_{0}} & = & c+\left(1-c\right)a(1-\tanh^{2}\left(a_{t}h_{0}\right)) \end{array}$$ (16)

$$\begin{array}{ccc} & = & c+(1-c)a, \end{array}$$ (17)

which shows that (*x*_0_, *h*_0_) is stable for *a* ∈ [0, 1[ and unstable for *a* > 1.

It follows that for *a* < 1, the system has a unique stable equilibrium point at (*x*_0_, *h*_0_), whose uniqueness is verified by the monotonicity of *G*(*h*_*t*_) (*dG*(*h*_*t*_)/*dh*_*t*_ > 0∀*h*_*t*_).

For *a* > 1, the point (*x*_0_, *h*_0_) is unstable, and there exist two stable points (*x*_0_, ±*h*_1_) whose basins of attraction are defined by *h*_*t*_ ∈ ]−∞, *h*_0_[ for −*h*_1_ and *h*_*t*_ ∈ ]*h*_0_, + ∞[ for *h*_1_.
